# Avian Hepatitis E Virus: With the Trend of Genotypes and Host Expansion

**DOI:** 10.3389/fmicb.2019.01696

**Published:** 2019-07-24

**Authors:** Peng Sun, Shaoli Lin, Shenghu He, En-Min Zhou, Qin Zhao

**Affiliations:** ^1^School of Agriculture, Ningxia University, Yinchuan, China; ^2^Department of Preventive Veterinary Medicine, College of Veterinary Medicine, Northwest A&F University, Yangling, China; ^3^Scientific Observing and Experimental Station of Veterinary Pharmacology and Diagnostic Technology, Ministry of Agriculture, Yangling, China; ^4^Division of Immunology, Virginia-Maryland Regional College of Veterinary Medicine, University of Maryland, College Park, College Park, MD, United States

**Keywords:** avian HEV, big liver and spleen virus, hepatitis-splenomegaly syndrome, hepatic rupture hemorrhage syndrome, genotypes, virology

## Abstract

Avian hepatitis E virus (HEV) is a single-stranded, positive-sense RNA virus with a complete genome of approximately 6.6 kb in size. To date, four major genotypes of avian HEV have been identified and classified into the *Orthohepevirus B* genus of the family *Hepeviridae*. The avian HEV associated with hepatitis-splenomegaly syndrome, big liver and spleen disease or hepatic rupture hemorrhage syndrome in chickens is genetically and antigenically related to mammalian HEV. With the increased genotypes of avian HEV identified, a broader host tropism is also notable in the epidemiological studies. Due to the lack of an efficient cell culture system, the mechanisms of avian HEV replication and pathogenesis are still poorly understood. The recent identification and characterization of animal strains of avian HEV has demonstrated the virus’ ability of cross-species infection. Although it has not yet been detected in humans, the potential threat of a zoonotic HEV capable of transmission to humans needs to be taken into consideration. This review article focuses on the current knowledge regarding avian HEV in virology, epidemiology, pathogenesis, clinical presentation, transmission, diagnosis and prevention.

**HIGHLIGHTS:**

- The mechanisms of avian HEV replication and pathogenesis are still poorly understood due to the lack of an efficient cell culture system.

- A broader host tropism is also notable in the epidemiological studies with the increased genotypes of avian HEV identified.

- The recent identification and characterization of animal strains of avian HEV has demonstrated the virus’ ability of cross-species infection.

- The potential threat of a zoonotic HEV capable of transmission to humans needs to be taken into consideration.

## Introduction

### History

In the early 1980s, big liver and spleen disease (BLS) was firstly characterized in chickens in Australia, and was considered to be the most significant disease affecting commercial broiler breeder flocks (Handlinger and Williams, 1988). It was clinically described as a sudden reduction in egg production, splenomegaly, hepatomegaly, and increased mortality (Handlinger and Williams, 1988). The causative agent of this disease, big liver and spleen virus (BLSV), was isolated in the late 1990s, and based on sequence analysis of a 523 bp nucleic acid fragment, it was found that this sequence of BLSV had a 62% homology with the helicase gene of human hepatitis E virus (HEV) (Payne et al., 1999; Zhao et al., 2017). Not long after, another disease affecting chickens, hepatitis-splenomegaly syndrome (HSS) was first described in British Columbia, Canada in 1991 and in eastern Canada and the United States in 1997 (Ritchie and Riddell, 1991; Riddell, 1997). It is characterized by pale to white combs with red edges, abdomens with red ascites, poor to moderate body condition, the regression of ovaries and oviducts, enlarged livers and spleens and increased mortality in 32-week-old broiler breeders and commercial egg laying hens (Riddell, 1997). In 2001, the HEV-related virus, named avian HEV, was characterized from HSS afflicted chickens in the United States. Based on genomic sequencing, this novel virus displayed approximately 50 and 80% nucleotide (nt) sequence identity with mammalian HEV and BLSV, respectively, suggesting that BLS and HSS are caused by variants of the same avian HEV (Haqshenas et al., 2001; Huang et al., 2002). Phylogenetic analyses indicated that avian HEV belongs to a separate floating genus within the family *Hepeviridae* (Meng, 2008; Yugo and Meng, 2013). In addition, in 2004, avian HEV isolated from presumed healthy flocks was shown to cluster with BLSV and form a distinct branch, which indicated these two are variant strains of the same virus (Sun et al., 2004).

To date, avian HEV is considered as a major causative agent of both BLS and HSS in chickens based on these findings. Avian HEV has now been detected in chickens in a number of countries, including the United States, Hungary, Canada, Australia, Spain, China, Korea, Poland, Israel and the cross-border region Austria and Czechia (Haqshenas et al., 2001; Agunos et al., 2006; Morrow et al., 2008; Bilic et al., 2009; Peralta et al., 2009; Marek et al., 2010; Zhao et al., 2010; Kwon et al., 2012; Konicek et al., 2016).

### Classification

Avian HEV belongs to the *Orthohepevirus B* species of the family *Hepeviridae* (Emerson and Purcell, 2003). The family contains two genera: *Orthohepevirus* (all mammalian and avian HEV isolates) and *Piscihepevirus* (cutthroat trout virus) (Smith et al., 2014; Spahr et al., 2018). There are four species within the genus *Orthohepevirus*: *A* to *D*. *Orthohepevirus A* consists of the strains isolated from human, pig, wild boar, deer, mongoose, rabbit, moose, and camel (Li et al., 2005; Kim et al., 2011; Izopet et al., 2012; Lee et al., 2016; Meng, 2016; Spahr et al., 2018). Within *Orthohepevirus A*, there are eight genotypes (1 to 8). Genotypes 1 and 2 are obligate human pathogens (Meng, 2013), whereas genotypes 3 and 4 can infect both human and animals (swine, rabbits, deer and mongooses) (Sridhar et al., 2017). Genotypes 5 and 6 are isolated from wild boars (Takahashi et al., 2011), and genotypes 7 and 8 are found in dromedary and Bactrian camels (Woo et al., 2014; Sridhar et al., 2017). *Orthohepevirus B* includes the viruses isolated from chickens and wild birds and named avian HEV (Meng, 2016; Reuter et al., 2016). *Orthohepevirus C* is composed of the strains isolated from rat, greater bandicoot, Asian musk shrew, ferret and mink (Meng, 2016), and *Orthohepevirus D* is isolated from bat (Meng, 2016). The zoonotic characteristic of HEV has raised great public health concerns regarding cross-species transmission and new genotype discovery in recent years (Mushahwar, 2008; Meng, 2010, 2011).

**FIGURE 1:**
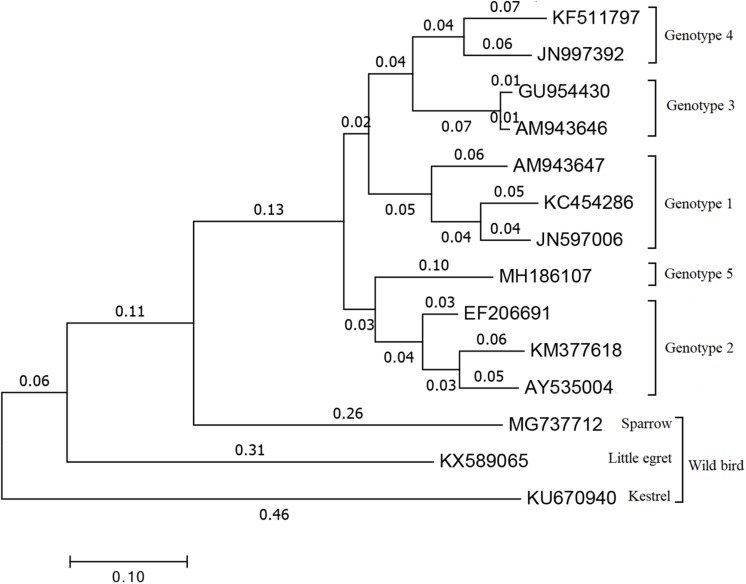
Phylogenetical tree of the current genotypes of different avian HEV strains based on the complete or near complete genomes. The tree was built by MEGA 7.0 using the neighbor-joining (NJ) method with 1000 bootstrap replicates. GenBank accession numbers represents different HEV strains. Genotype 1: AM943647 is for avian HEV strain in Australia, JN597006 and KC454286 are for avian HEV strain in Korea. Genotype 2: AY535004 and EF206691 are two U.S. avian HEV strain, KM377618 is avian HEV strain in Korea. Genotype 3: AM943646 represents European avian HEV strain and GU954430 represents Chinese avian HEV strain. Genotype 4: JN997392 is for avian HEV strain in Hungary and KF511797 is for avian HEV strain in Taiwan region. Genotype 5: MH186107 stands for the recent new type avian HEV strain which isolated in Hebei, China. MG737712 is for sparrow, KX589065 is for little egret and KU670940 is for kestrel which are all representing avian HEV strain isolated from wild birds.

Within *Orthohepevirus B*, different avian HEV strains share approximately 73 to 100% identity to one another, and approximately 30 to 50% identity with mammalian HEVs (Huang et al., 2002). Although avian HEV only shares ∼50% identity to human and swine HEV strains, the genomic organization is relatively conserved (Haqshenas et al., 2001; Huang et al., 2004). Now, four major genotypes (1 to 4) were distinguished by genome-wide sequencing (Figure 1) and share approximately 82% identity at the nucleotide level (Syed et al., 2017). Genotype 1, was primarily isolated from BLS chickens in Australia and was also reported to cause the decrease in egg production in chickens during an outbreak in Korea (Payne et al., 1999; Kwon et al., 2012). Genotype 2, was primarily isolated from HSS chickens in the United States and was detected in BLS flocks in Central Europe and Poland, and also isolated from chickens with hepatitis-splenomegaly syndrome in Korea (Haqshenas et al., 2001; Moon et al., 2016; Matczuk et al., 2019). Genotype 3, was isolated from HSS chickens in Europe and from hens with decreased egg laying in China (Morrow et al., 2008; Zhao et al., 2010). Genotype 4, was isolated from affected flocks in Hungary and from bile samples of commercial egg-layer flocks in Taiwan region, which were initially presumed to be healthy (Bányai et al., 2012; Hsu and Tsai, 2014). Recently, a new isolate from chickens suffering from hepatic rupture hemorrhage syndrome (HRHS) in China was shown to be associated with avian HEV and named avian HEV-Hebei, phylogenetic analyses constructed according to the partial *helicase* and partial *capsid* gene showed in a single branch in avian HEV groups but distinctly separate from other avian HEV strains, which may further extend the genotypes of avian HEV (Figure 1) (Su et al., 2018a, b). This isolate belongs to a single branch, distinctly separated from other reference avian HEV strains (Figure 1). While all of these genotypes tend to be geographically different, each genotype of avian HEV belongs to a single serotype (Marek et al., 2010; Meng, 2010; Zhao et al., 2015).

## Virology

### Virion Properties

Avian HEV is a single-stranded, positive-sense RNA virus which is sensitive to high temperature and iodinated disinfectants, but with certain resistance to acidic and mild alkaline conditions (Meng, 2010). The virions are icosahedral and symmetrical with a diameter of approximately 30–35 nm in size (Meng, 2010). The buoyant density of avian HEV is 1.39–1.40 g/cm^3^ in cesium chloride (CsCL) and 1.29 g/cm^3^ in potassium tartrate and glycerol according to a report by Meng (2010).

### Genome Organization

**FIGURE 2:**
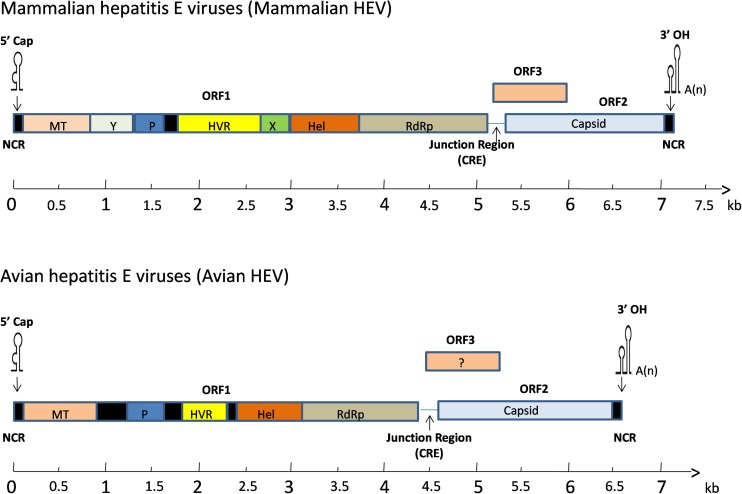
A schematic diagram of comparative genomic organization of mammalian and avian HEV. The three open reading frames (ORFs) are labeled with different colors and shown as above. The avian HEV genome is approximately 600 bp smaller than the mammalian HEV. ORF1 encodes non-structural proteins with the putative functional domains. ORF2 encodes the capsid protein, and ORF3 encodes a phosphoprotein. The ORF2 and ORF3 are partially overlapped, but neither overlaps with ORF1. The genome is capped at the 5′ end and contains a poly(A) tail at the 3′ end. The open reading frame is flanked by non-coding regions (NCR) at the 5′ and 3′ ends of the genome. A junction region exists between ORF1 and ORF3 for mammalian and avian HEV, which is characterized with a stem-loop structure and a cis-reactive element (CRE). MT, methyltransferase; P, a papain-like cysteine protease; HVR, hypervariable region; Hel, helicase; RdRp, RNA-dependent RNA polymerase.

The full-length genome of avian HEV is ∼6.6 kb in length, which is 600 bp shorter than mammalian HEV (Figure 2). It contains a short 5′non-coding region (NCR), followed by three open reading frames (ORFs) and a short 3′NCR terminated by a poly(A) tract (Kabrane-Lazizi et al., 1999). The ORF2 and ORF3 are partially overlapped, but neither overlaps with ORF1 (Meng, 2010). The length of the 5′NCR of avian HEV is 24 bp, which is 2–4 bp shorter than that of most human and swine HEV (Huang et al., 2004). The ORF1 (nt 25 to 4620) of avian HEV encodes a viral non-structural protein 1531 amino acids (aa) in length with multiple functional domains based on computer predictions, including a methyltransferase (Methyl, aa 56–241), papain-like cysteine protease (PLP, aa 433–593), hypervariable region (HVR, aa 673–802), helicase (Hel, aa 984–1216) and RNA-dependent RNA polymerase (RdRp, aa 1231–1720) (Huang et al., 2004, 2007; Bilic et al., 2009). The typical motifs I-VI of the helicase superfamily I and the motifs I-III of viral methyltransferase are found conserved throughout the alpha-like virus super-group (Huang et al., 2004).

ORF1 accounts for the largest portion in the genome and its products play a very important role in the proper assembly and release of virus particles (Ahmad et al., 2011). The ORF2 (nt 4707 to 6527) encodes the capsid protein of 606 aa in length, sharing 48–49% aa identity with swine and human HEVs (Haqshenas et al., 2001). Six linear antigenic domains (I–VI) have been characterized in avian HEV ORF2 protein, which are located in aa 389–410, 461–492, 556–566, 583–600, 339–389, and 23–85, respectively (Dong et al., 2011; Wang et al., 2014). Domains I and V contains epitopes, which are common among avian, human, and swine HEVs (Wang et al., 2015). The domains II and VI are unique to avian HEV and can induce immune responses in chickens, while domains III and IV only elicit a weak immune response (Zhao et al., 2015). Collectively, the capsid protein contains the most immunogenic epitopes capable of inducing neutralizing antibodies, which are a potential target for vaccine development (Meng, 2008). The smallest one, ORF3 (nt 4654 to 4917) encodes a protein of 87 aa (Huang et al., 2004). Three antigenic domains on ORF3 at amino acids (aa) 1–28, 55–74 and 75–88 were identified in the Chinese isolate of avian HEV (CaHEV) (Zhao et al., 2013b). The aa 75–88 region was shown to be a dominant antigenic domain which contains at least two epitopes, though the neutralizing characteristics of these three antigenic domains are still unknown (Zhao et al., 2013b). The downstream of ORF2 is the 3′NCR of avian HEV, excluding the poly(A) tail, consists of 123 nt (Bilic et al., 2009).

## Epidemiology

Avian HEV is considered to be the main causative agent of BLS and HSS (Haqshenas et al., 2001; Huang et al., 2002), which has been reported in Australia (Handlinger and Williams, 1988), the United States (Haqshenas et al., 2001), Canada (Ritchie and Riddell, 1991; Agunos et al., 2006) and Europe (Morrow et al., 2008). In addition, molecular epidemiologic investigations show high HEV-positive rates in chicken flocks in the United States, and that the virus can cause subclinical infections in these chickens (Sun et al., 2004). Serologic investigations show that the positive rate of chicken flocks and chickens are 71 and 30%, 57 and 28%, 95.08 and 40.57% in the United States (2002), Korea (2012) and throughout Taiwan (2013), respectively (Huang et al., 2002; Kwon et al., 2012; Hsu and Tsai, 2014). There is a 20% positive rate for anti-avian HEV antibodies in chickens from Brazil (Vitral et al., 2005), and in Spain, positive rates for farms span a wide range from 20 to 80% (Peralta et al., 2009). In China, the avian HEV seropositive rate in chicken flocks is 35.9% (Zhao et al., 2013a), while in Poland, there is a 56.1% positive rate for avian HEV genotype 2 among breeder broiler and egg laying hen flocks (Matczuk et al., 2019). In the United States between the years 2012 and 2014, 141 chickens from 10 organic layer flocks showed a 40% decrease in the rate of egg production and slightly increased mortality (up to 1% per week), avian HEV RNA was detected in 10 livers out of 141 chickens from two different affected flocks (Crespo et al., 2015). Recently, a disease known as HRHS was proven to be associated with avian HEV, and has emerged in many large-scale layers and breeding farms in China since 2016 (Su et al., 2018a). During this time, over 270,000 chickens from four farms in the Guangdong, Anhui, Hebei and Jilin provinces of China were positive for avian HEV. Those affected flocks showed several death peaks at ages 1 to 5 weeks, 17 to 20 weeks and 27 to 40 weeks. The cumulative mortality was approximately 15%, and the egg laying peak was significantly delayed with a laying rate decreased by 20% (Su et al., 2018b).

Whether infections with avian HEV are age-associated or not, it’s still known of ambiguity as there have been a number of conflicting results. One study showed that 17% of chickens less than 18 weeks old and 36% of adult chickens in the United States were positive for avian HEV antibodies using a truncated recombinant ORF2 antigen (Huang et al., 2002). On the other hand, another report on commercial egg-type chicken flocks in the United States, identified avian HEV in 17-week-old chickens but no infections in 30 to 80-week-old chickens (Gerber et al., 2014). A third study showed that high antibody titers in infected chickens can be expected within the ages of 20–29 weeks (Zhao et al., 2013a). Meanwhile, 10% of chickens at 60 weeks old were also found to be avian HEV seropositive (Zhao et al., 2013a). The result is variable, the different region and flocks may contribute to this issue. Thus, a systematic artificially infection experiment should carry out to confirm age-associate result.

In addition to chickens, avian HEV has also been identified in wild aquatic birds, such as the little egret in Hungary, which may represent a potential novel *Orthohepevirus* species (Reuter et al., 2016). An avian-like HEV from sparrow feces in the Midwestern United States has also been detected, which may also represent a novel HEV which is related to chicken and little egret HEVs (Yang et al., 2018). In both affected and presumed healthy poultry flocks across Russia and Kazakhstan, a high genetic divergence of avian HEV has also been detected (Sprygin et al., 2012). However, the avian HEV isolates from wild bird to cause chicken infection or the ones from chicken to cause wild bird infection are still uncertain (Reuter et al., 2016). Based on the phylogenetic tree, we speculate that the avian HEV strains from chicken and wild bird have an independent evolution from the same HEV ancestor. In addition, based on the comparisons between the different avian HEV isolates, they have the possible ability for cross-transmission (Meng, 2010).

Co-infection is common in clinical case, avian HEV co-infected with other pathogens has also been reported (Sun et al., 2016; Yang et al., 2016). For example, an epidemiology study showed that both avian HEV and avian leucosis virus subgroup J infection is endemic in those flocks with HSS in China (Sun et al., 2016). Additionally, avian HEV and Marek’s disease virus (MDV) was found to be co-prevalent in a flock of egg-type chickens in China with a double virus positive rate of ∼30% (Yang et al., 2016).

## Clinical Presentation

In chickens, subclinical infections of avian HEV are very common in flocks in the United States and other countries, but the morbidity and mortality associated with HSS (or BLS) and other avian HEV infections in the field are relatively low (Huang et al., 2002; Sun et al., 2004; Peralta et al., 2009). The mortality rate is between 0.3 and 1% for avian HEV genotypes 1–3 infections (Meng et al., 2008; Nikonova and Zinyakov, 2012; Yugo et al., 2016). The clinical presentation caused by the virus can be variable. Broiler breeders which have been inoculated with avian HEV genotype 1, typically show more gross ovarian and microscopic lesions than younger chickens inoculated with genotype 2, while avian HEV genotype 3 infection of laying hens can exhibit BLS or HSS, but shows decreased egg production without gross ovary and microscopic lesions (Billam et al., 2005; Park et al., 2015; Zhao et al., 2017). There are several other diseases that could be caused by avian HEV, which are listed as follows (Table 1).

**TABLE 1:** Comparison of three different diseases caused by avian HEV.

**Disease**	**Clinical symptoms**	**Pathology**	**Outcome Results**
BLSV (Handlinger and Williams, 1988)	1. pallor of the combs 2. Anorexia 3. Drowsiness 4. Pasty droppings and soiling of the vent feathers 5. Hens found dead usually have a good body condition. 6. Enlarged and mottled livers and spleens 7. Ovaries are regressing 8. Peritonitis with free yolk in the abdominal cavity 9. Petechial hemorrhage of the duodenum 10. Swollen kidney 11. Edema of the lung is rare	1. There are occasional numerous small and white foci scattered among the red pulp, showing large areas of hemorrhage and necrosis in the liver and spleen. Ovaries with blood clots often present within the large, pale, flaccid follicles. 2. There are five stages of splenic and hepatic pathology according to Handlinger’s report: I. Lymphoproliferative phase. II. Pyknotic destructive phase. III. Macrophage responsive phase. IV. Late responsive phase. V. Recovered. 3. In the early stage of the disease, lymphocytes and heterophilic cells infiltrate around the portal area of the liver 4. Lymphocytes proliferate in the spleen 5. Coagulative necrosis and vasculitis occur in the liver.	1. A rapid and sudden drop in egg production last for 3–4 weeks, following by a return to normal production after another 3 weeks. 2. The mortality rate increased from 0.1–1% per week. 3. The signs come out as early as 24 weeks or late than 58 weeks age, production rate decreased by 5–20%.
HSS (Handlinger and Williams, 1988; Tablante et al., 1994; Huang et al., 2002; Meng, 2011)	1. Ovarian degeneration 2. Abdominal redness 3. Liver is amyloid or fatty degeneration with occasionally hepatosplenomegaly.	1. Subcapsular hemorrhages and slight swelling of the liver lobes. 2. The microscopic liver lesions were mainly lymphocytic, heterophilic periphlebitis and phlebitis with occasional biliary vacuolation, amorphous hypocellular eosinophilic matrixes, hemorrhages, and necrotic foci.	1. 20–40% egg production rate of hens 2. Broiler breeders at 30–72 weeks (increased 1%death rate), with mortality lasting several weeks during the midproduction period. 3. Higher incidence of chickens at 40–50 weeks.
HRHS (Su et al., 2018a, b)	1. Severe depression with pale crest 2. Doiling of the vent feathers 3. Red fluid in the abdomen.	1. Hemorrhage in the liver and spleen, while no obvious pathological changes in other organs.	1. The death peak of sick flocks is at age of 1 to 5 weeks, 17 to 20 weeks and 27 to 40 weeks. 2. The mortality is approximately 15%, and the laying peak is significantly delayed with the rate drop by 20%.

## Pathogenesis

Considering the economic losses and the increased clinical infection rates, avian HEV pathogenesis research has become a necessity. The current method to study avian HEV pathogenicity is limited since the virus cannot be efficiently propagated in cell culture, and can only proliferate within living organisms or animals. As such, the pathogenicity of avian HEV remains largely uncharacterized (Meng, 2010; Syed et al., 2017).

Billam et al. (2005) infected 60-week-old specific-pathogen-free (SPF) adult chickens by a natural route, to systematically study HEV pathogenesis and replication in a homologous animal model. They found avian HEV RNA in both the bile and liver of infected chickens, indicating that the virus replicates in the liver. Billam et al. (2008) showed that viral replication initially takes place in gastrointestinal tissues including the colorectal, cecal, jejunal, ileal, duodenal, and cecal tonsil tissues prior to reaching the liver (Billam et al., 2008; Yugo et al., 2016). Based on these results, it can be inferred that the entry of avian HEV first occurs from an oral-route, and then replicates and accumulates in the digestive system. In addition to viral replication, virus attachment to host cells is considered to be another crucial step in viral infections (Schneider-Schaulies, 2000; Yugo et al., 2016). It is reported that the capsid protein region from amino acid 471 to 507 is critical for avian HEV attachment to host cells (Zhang et al., 2016) and the C-terminal region of the capsid ORF2 protein is recognized as putative binding sites for both cellular receptors and neutralizing antibodies (Ahmad et al., 2011). Wang et al. (2018) utilized *Lactococcuslactis* as a delivery vector expressing higher levels of ΔORF2 (avian HEV truncated ORF2 protein spanning amino acids 249–606 protein) specific IgG antibodies, in order to neutralize or bind to avian HEV and induce effective mucosal immunity, prevented the virus from invading into host cells. These results also showed effective control of avian HEV infection in chickens. Better understanding of viral pathogenesis and replication both *in vivo* and *in vitro* is crucial for controlling and diminishing avian HEV infections.

## Transmission

Avian HEV is believed to be transmitted mainly by the fecal-oral route through contaminated feed and water in different flocks (Haqshenas et al., 2002; Huang et al., 2002). Furthermore, another new route of transmission has been successfully identified by Billam et al. (2005) experiment in which avian HEV infection was successfully reproduced via nasal and oral route inoculation in SPF chickens. To determine whether avian HEV can be transmitted vertically, Guo et al. (2007) collected and tested embryonated eggs starting from 1 week prior to virus inoculation to 5 weeks post-infection (p.i.), but all of the hatched chicks were negative for avian HEV infection. Conversely, Troxler et al. (2014) research, demonstrated that avian HEV RNA could be detected at week 25 in chicks hatched from laid eggs, indicating the possibility of vertical transmission routes.

Within the eight species of *Orthohepevirus A*, genotypes 1 and 2 exclusively infect humans, whereas genotypes 3, 4, 7 and 8 can cross species barriers and are denoted as zoonotic genotypes (Purcell and Emerson, 2008; Doceul et al., 2016; Lee et al., 2016; Spahr et al., 2018), and according to the very recent study, *Orthohepevirus C* was identified to be zoonotic (Murphy et al., 2019). However, *Orthohepevirus A* (genotypes 5, 6) and *D* have not been labeled as zoonotic diseases (Liu et al., 2018). Surprisingly, the avian HEV (a member of *Orthohepevirus B*) also can be transmitted across species barriers, though they do not infect rhesus monkeys or pigs (Sun et al., 2004), but has been reported to infect turkeys under experimental conditions (Sun et al., 2004) and as well as other birds under natural conditions (wild aquatic bird, little egret, song thrush, little owl, feral pigeon and common buzzard), suggesting that avian HEV may have a limited host range and may not infect humans, and that chickens are likely not a reservoir for avian HEV (Huang et al., 2004). Meanwhile, genotypes 1 and 3 are prevalent in wild birds of 4 different species (song thrush, little owl, feral pigeon and common buzzard), which indicates avian HEV transmission between chickens and wild birds (Meng, 2010; Zhang et al., 2017). The presence of HEV in wild birds indicates that wild birds might play a role as a reservoir for avian HEV (Zhang et al., 2017). In addition to wild birds and turkeys, a mixed animal group comprised of chickens, ducks, geese and rabbits also can be infected with avian HEV genotype 3, demonstrating avian HEV could be a cross-species transmission pathogen (Liu et al., 2018). Though avian HEV has not been reported to cause zoonotic infections, its expanded host range and its ability to infect across species raises additional concerns (Meng, 2010).

For the mammalian HEV, it was reported that the ORF1 is considered to play an indispensable role in determining host tropism (Chatterjee et al., 2016). However, there were also some reports which HEV ORF2 and ORF3 protein, host factors, especially host immune status, may also minorly contribute to cross-species transmission of HEV (Ding et al., 2017). For avian HEV, it was also documented that more mutated amino acids in ORF1 of the avian HEV isolated from the rabbit, suggesting that ORF1 maybe also play a role in the cross-transmission of avian HEV (Liu et al., 2018). However, the underlying molecular mechanism that ORF1 plays to alter host cell tropism during HEV cross-species infection is not yet clear.

## Diagnosis

The diagnosis for clinical manifestation of avian HEV is based on the detection of specific viral RNA, as the clinical signs and pathologic lesions are atypical (Crespo et al., 2015). All presumptive diagnoses must rely on laboratory tests, primarily based on RT-PCR for virus detection, enzyme-linked immunosorbent assay (ELISA) for avian HEV ORF2 and ORF3 specific IgG antibody detection and electron microscope (EM) for virus particles detection (Meng, 2010). Other lesion detection markers such as serum lactate dehydrogenase (LDH) and serum alanine aminotransferase (ALT) could possibly be used as a supplementary diagnostic method since both are shown to be increased after intravenous (i.v.) and oronasal inoculation.

Avian HEV-specific RT-PCR assays have been developed for the detection of avian HEV infections in chickens (Sun et al., 2004). The nested RT-PCR assays were universally used to detect avian HEV RNA from the fecal, serum, bile, liver and spleen samples. To determine avian HEV infection in the chickens, two truncated genes (ORF1 and ORF2) were amplified by nested RT-PCR at the same time. Recently, the pair of primers designed by Sun et al. (2004) were universally detected different genotypes of avian HEV RNA. Compared with conventional RT-PCR, duplex TaqMan real-time RT-PCR assay was shown to be highly specific (Troxler et al., 2011). A SYBR Green real-time RT-PCR assay was developed for the rapid, specific, and reproducible diagnosis of avian HEV infection in chickens by Zhao et al. (2015), which can accurately detect avian HEV RNA in serum, liver, spleen, and fecal samples with more sensitivity than conventional RT-PCR.

The indirect ELISA (iELISA) methods were also improved, using a newly developed truncated recombinant ORF2 antigen of avian HEV genotype 2 strain to determine avian HEV seroprevalence (Huang et al., 2002). Zhao et al. (2013b) used a truncated ORF2 protein from an avian HEV genotype 3 strain isolated in China (CaHEV) as the coating antigen, and got a sensitivity of 96.1% and a specificity of 95.8%, with 97% corresponding with Western blot results, which can be used for the detection of chicken immunoglobulin G antibodies (IgG) against avian HEV for Chinese poultry farms. The blocking enzyme-linked immunosorbent assay (bELISA) had a sensitivity of 98.3% and a specificity of 93.3% with CaHEV and had no cross-reaction with other anti-avian virus antibodies which can be used for detection of antibodies against avian HEV and showed high reproducibility compared with indirect ELISA and Western blot methods (Liu et al., 2014). Furthermore, two indirect ELISAs, using the truncated capsid proteins separately from the prototype avian HEV genotype 2 and genotype 3 as coated antigens, can be used effectively for the sero-epidemiological detection (Huang et al., 2002). Generally, the C-terminal region of capsid protein (268 amino acids) expressed by bacterial system were used as the coated antigen in ELISA assay (Haqshenas et al., 2002). Four antigenic domains were identified from the region (Zhao et al., 2015). Out of which, the domain I (aa 389–410) and domain V (aa 339–389) can induce a strong immune response in chickens, so this is the optimal selected domain as an antigen for detection of antibodies against avian HEV (Zhao et al., 2014). In addition, the ORF3 protein with lower sensitivity and specificity comparing with ORF2 protein was also used as the antigen to detect antibodies against avian HEV (Syed et al., 2017).

However, the specificity and consistency of the ELISA assays using the different genotypes avian HEV as coating antigen is unknown, since avian HEV isolates from different geographic regions are genetically heterogenic (Huang et al., 2002; Zhao et al., 2013b). Most of these assays are based on antigens expressed by a single avian HEV genotype; these might be a limitation for detecting all other avian HEV genotypes (Zhao et al., 2013b). To efficiently detect HEV infection from different sources, the identification of conserved epitopes may facilitate the development of diagnosis kits for region-specific avian HEV detection.

## Prevention and Control

Avian HEV infection is common in chicken flocks, however, there is no effective measure to prevent the disease from spreading (Reuter et al., 2016). Due to the lack of effective commercial vaccines and drugs for preventing disease in chickens, blocking fecal-oral transmission should prevent the spread of virus infection, while the implementation of strict biosecurity regulations at chicken farms may limit the spread of the virus (Molaei et al., 2006; Meng, 2010; Zhao et al., 2017). From Liu et al. (2017) study, cage-free compared to caged animals showed higher positive rates for both antibodies and RNA of avian HEV. The control of chicken excrement pollution could reduce avian HEV infection while implementing a caged living/raising method can help to better prevent HEV transmission. Conversely, Sun et al. (2016) believed epidemiological investigation and elimination of infected chickens to be the only efficient methods for avian HEV prevention and control due to the subclinical and persistent infections in chickens and the lack of availability of vaccines or treatment options (Sun et al., 2016; Nimgaonkar et al., 2017). The most effective measures for avian HEV prevention and control should combine two methods in clinical work since it’s the core of biosecurity-based process.

Truncated avian HEV ORF2 contains immunodominant epitopes, which can induce a protective humoral immune response (Schofield et al., 2000; Meng et al., 2001). Guo et al. (2007) showed that recombinant ORF2 epitope identification can promote the development of efficient vaccines. While the complete ORF3 proteins were evaluated for immunoprotection of chickens against CaHEV infection, Syed et al. (2017) proved the expression of ORF3 protein only provides partial immune protection.

Most of these recombinant ORF2/ORF3 products depend on an *Escherichia coli*-bacterial expression system (Dong et al., 2011; Zhao et al., 2013b), while Wang et al. (2018) utilized *Lactococcuslactis* as a delivery vector for truncated avian HEV ORF2 protein, and demonstrated that avian HEV ORF2 protein can induce ideal protection against hepatitis and liver injury caused by avian HEV, indicating that vaccine production using different vectors may differentially alter host immune responses.

## Conclusion and Future Perspectives

HEV is an important pathogen affecting both humans and animals, yet many questions remain unanswered, and more research needs to be done to better understand pathogenesis and to aid in the development of vaccines and treatments (Meng, 2010; Yugo et al., 2016). A vaccine for human use, based solely on the capsid protein of a genotype 1 HEV isolate, named Hecolin^®^ , has been approved in China (Nan et al., 2018), but commercial vaccine for avian HEV is unavailable. Though avian HEV is not currently considered a zoonotic disease, it possesses cross-species infection ability with wide prevalence in most countries, which has aroused more attention for avian HEV prophylaxis and treatment. Vaccine development using synthetic peptides against avian HEV may help to minimize the risks of cross-species transmission and limit potential food borne transmission routes (Meng, 2010). The detection of avian HEV is based on the capsid protein (contains immunodominant epitopes and induces a protective humoral immune response) and antigenic domains for laboratory avian HEV serum antibody, but not all these antigens are commercially available (Syed et al., 2017; Nan et al., 2018). Further epitope identification can improve the accuracy of diagnosis kit and the efficacy of subunit vaccines (Nan et al., 2018). Additional pathogenesis studies on the virus could also provide new insights to combat HEV infection. With the accumulation of these fundamental works, it is promising that upcoming avian HEV vaccines will successfully protect against avian HEV, a broad cross-species virus.

## Author Contributions

All authors listed have made a substantial, direct and intellectual contribution to the work, and approved it for publication.

## Conflict of Interest Statement

The authors declare that the research was conducted in the absence of any commercial or financial relationships that could be construed as a potential conflict of interest.
